# Stranded dolphin stomach contents represent the free-ranging population's diet

**DOI:** 10.1098/rsbl.2012.1036

**Published:** 2013-06-23

**Authors:** Glenn Dunshea, Nélio B. Barros, Elizabeth J. Berens McCabe, Nicholas J. Gales, Mark A. Hindell, Simon N. Jarman, Randall S. Wells

**Affiliations:** 1 Institute of Marine and Antarctic Studies, University of Tasmania, Sandy Bay, Tasmania 7005, Australia; 2 Australian Marine Mammal Centre, Australian Antarctic Division, Channel Highway, Kingston, Tasmania 7050, Australia; 3 Chicago Zoological Society c/o Mote Marine Laboratory, 1600 Ken Thompson Parkway, Sarasota, FL 34236, USA

**Keywords:** DNA-based, PCR, Sarasota, foraging

## Abstract

Diet is a fundamental aspect of animal ecology. Cetacean prey species are generally identified by examining stomach contents of stranded individuals. Critical uncertainty in these studies is whether samples from stranded animals are representative of the diet of free-ranging animals. Over two summers, we collected faecal and gastric samples from healthy free-ranging individuals of an extensively studied bottlenose dolphin population. These samples were analysed by molecular prey detection and these data compared with stomach contents data derived from stranded dolphins from the same population collected over 22 years. There was a remarkable consistency in the prey species composition and relative amounts between the two datasets. The conclusions of past stomach contents studies regarding dolphin habitat associations, prey selection and proposed foraging mechanisms are supported by molecular data from live animals and the combined dataset. This is the first explicit test of the validity of stomach contents analysis for accurate population-scale diet determination of an inshore cetacean.

## 1. Introduction

Knowledge of diet is a foundation of consumer ecology and fundamental to empirical investigations of food webs, competition, consumer evolution and ecosystem dynamics. Gathering diet information for marine mammals is particularly challenging owing to their life habits and the inability to observe feeding without bias [1]. Unlike studies of marine mammals that use terrestrial habitat, cetacean diet studies are further constrained by difficulty in obtaining samples. Many contemporary investigations of cetacean prey employ stomach contents analysis (SCA) of samples gathered opportunistically from fisheries by-catch and/or stranded carcasses, which yields information on prey species, size and relative composition [1–3].

Despite the insights gained from opportunistic SCA, there is uncertainty whether these data are representative of healthy free-ranging populations [3–5]. SCA also has well-recognized biases, such as under-representation of prey lacking robust hard parts [5]. There has been limited ability to investigate potential confounding effects of these facets of SCA on past cetacean diet studies. Ideally, this would involve sampling healthy free-ranging animals and the use of independent comparable methods, but most non-lethal methods, such as stable isotope or fatty acids analysis lack the prey taxonomic resolution of SCA. While high taxonomic resolution of live pinniped diet is possible via faecal hard parts analysis, cetacean faeces do not contain visually diagnostic prey remains. However, identification of prey in cetacean faeces is possible using molecular techniques [6,7], which are sensitive and only require DNA fragments to survive the digestion process [8].

The foraging ecology of resident bottlenose dolphins (*Tursiops truncatus*) in Sarasota Bay, FL, USA, has been thoroughly studied by SCA, prey abundance and distribution investigations and experimental evaluations of proposed foraging mechanisms [1,2,9]. This has been facilitated by extensive long-term research of the resident population, which includes an occasional health assessment programme [10]. The latter programme facilitated collection of faecal and gastric samples from healthy free-ranging dolphins. We analysed these samples using molecular prey detection techniques and compared these data with SCA data from stranded animals with the aim of determining whether the SCA results accurately describe the diet of live dolphins.

## 2. Material and methods

Faecal (*n* = 15) and gastric (*n* = 9) samples were collected from 19 live resident dolphins in June 2005 and 2006. Faecal samples were collected in 50 ml falcon tubes directly from the animals when they defecated while being handled for the purposes of the health monitoring programme (see [10] for capture and handling procedures). In most cases, samples were collected straight from the anus of the defecating dolphin on deck of the research vessel, however, in 2006 three samples were scooped from the water in 50 ml falcon tubes, where the dolphins were being handled and sea water was drained by fastening the tube cap loosely and inverting. Samples were stored on ice until addition of one volume of 100 per cent ethanol, with further storage at −20°C until analysis. Gastric samples were collected by inserting a 10 mm plastic hose down the oesophagus. The hose was passed down to the fore-stomach and gastric juice drained off into a sterile 50 ml falcon tube, which was sub-sampled into sterile 15 ml falcon tubes, one of which was used for this study. These samples were stored at −80°C until analysis.

Molecular prey detection using these samples was performed using the methods of Dunshea [6] where DNA was extracted, prey mitochondrial DNA PCR amplified and cloned, clone libraries sampled and clone inserts sequenced. Prey sequences were identified by reference to GenBank sequences and using Bayesian phylogenetic analysis [11]. All sample metadata and details of molecular and bioinformatic procedures are given in the electronic supplementary material. Prey proportions in DNA samples were inferred from proportions of prey amplicons in clone libraries pooled within individuals and averaged between them, as an indication of relative prey quantity [12]. Results from samples where no prey DNA was detected were discarded (*n* = 1).

Sarasota Bay resident dolphin SCA data from past studies [1,2] were collated and pooled ignoring individuals with empty stomachs (*n* = 32). Mean numerical abundances of SCA data were compared with mean clone proportions. Prey absolute frequency of occurrence (FOC) and exact binomial confidence intervals (*sensu* [13]) were compared between SCA and DNA data as were sample-based rarefaction curves for prey diversity discovery calculated by the Mau Tau method using EstimateS software [14]. Both the presence/absence of prey and numerical/clone per cent composition were tested for similarity between the two datasets by analysis of similarity (ANOSIM) with the Bray–Curtis similarity measure and 9999 permutations, testing for significant differences between SCA and DNA data with PRIMER software [15].

Routines to compare datasets were performed in duplicate where data from samples collected from the water column were excluded, to examine the effect of potential meroplankton contamination in these samples. Excluding these samples did not affect any outcome (the full analysis is presented), and we consider potential meroplankton contamination to be minimal or consistent, given the similarity of results from these samples to all other faecal samples.

## 3. Results

A total of 447 clones was screened from 23 clone libraries (15–38 clones per library) created from faecal and gastric samples from 18 live dolphins. Excluding dolphin mtDNA and nuclear mitochondrial pseudogene sequences [16], 355 clones of 53 unique sequences formed 32 prey molecular operational taxonomic units (MOTUs: see the electronic supplementary material) that were further grouped into 27 taxonomic identifications consisting of 14 species, nine genera and four family-level identifications, from a total of 16 teleost families, with 3.6 ± 1.7 prey taxa per sample (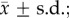 range 1–7). Chimeric prey sequences were detected in two samples and these were discarded. MOTU, taxonomic identification and GenBank submission details for all unique sequences are provided in the electronic supplementary material.

The stomach contents from 32 stranded dolphins with prey present consisted of 544 prey items (range 1–92 per dolphin). Thirty six prey taxa were discriminated, consisting of 20 species, 11 genus, five family and one subclass-level identification, from 19 teleost families, one family of squid and an elasmobranch, with 3.4 ± 2.9 prey taxa per sample (range 1–14).

Combining the molecular and stomach contents datasets required standardization of some prey categories owing to taxonomic resolution inconsistencies between methods. Species were grouped into the genera *Brevoorita*, *Cynoscion*, *Elops*, *Opsanus*, *Opisthonema* and *Sphoeroides* (table 1). SCA data were not grouped into family-level MOTU taxonomic assignments since database sequences were present for the SCA species within these families in most cases. Given the aim of this study, the more conservative approach was to leave these MOTUs as separate prey categories, despite the fact that they may represent the same items.

**Table 1.** Frequency of occurrence (FOC) of prey taxa identified in this study using stomach contents analysis (SCA) and molecular prey detection (DNA) and pooled numerical (SCA) or amplicon (DNA) proportions. Grey shaded rows are prey identifications from either method that may represent taxa identified by the other method.

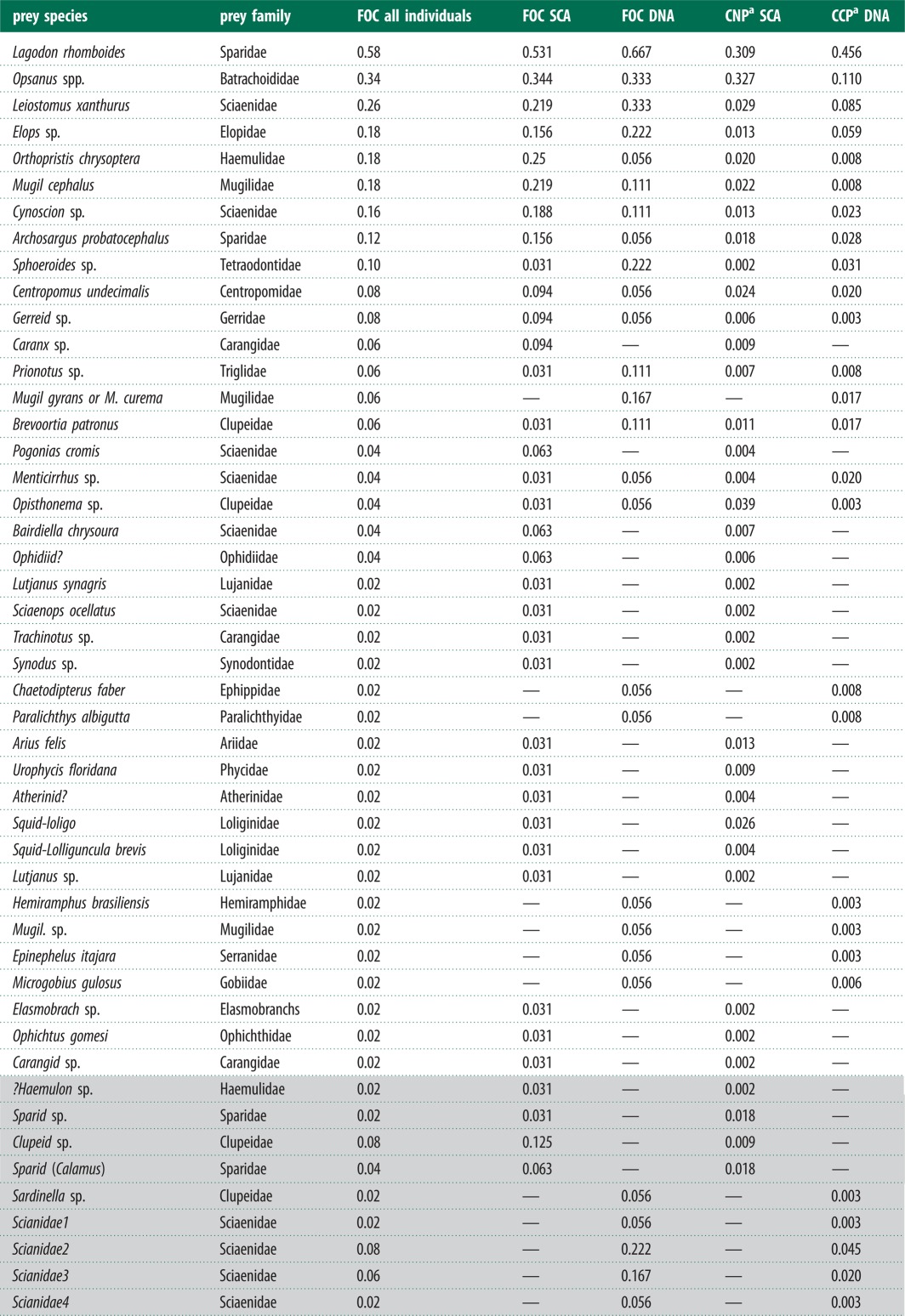

**Figure 1.**
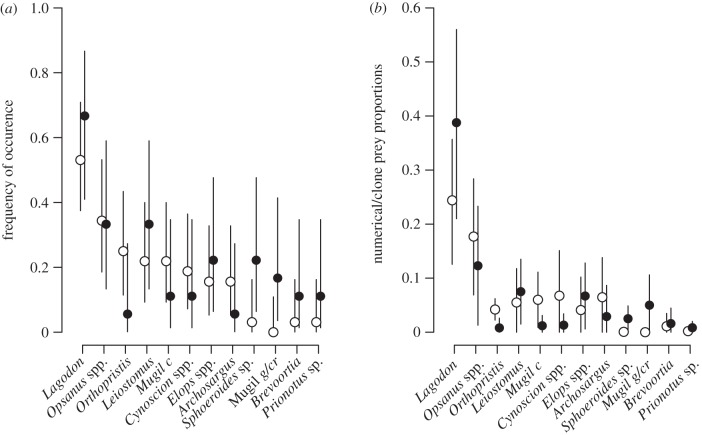
Comparison of prey metrics between stomach contents analysis of stranded dolphins (SCA, open circles) and molecular prey detection in samples from live animals (DNA, closed circles) from either method where prey frequency of occurrence (FOC) was more than 10%. (*a*) FOC with exact binomial 95% confidence intervals (CIs). (*b*) Mean numerical (SCA) or amplicon (DNA) prey proportions and truncated 95% CIs. *x*-axis labels are unique prey names (genus/genus sp. initials): see table 1 for details.

^a^The combined numerical proportion (CNP) or combined clone MOTU proportion (CCP) pooling data from all samples. See figure 1 for mean proportions across samples.

**Figure 2.**
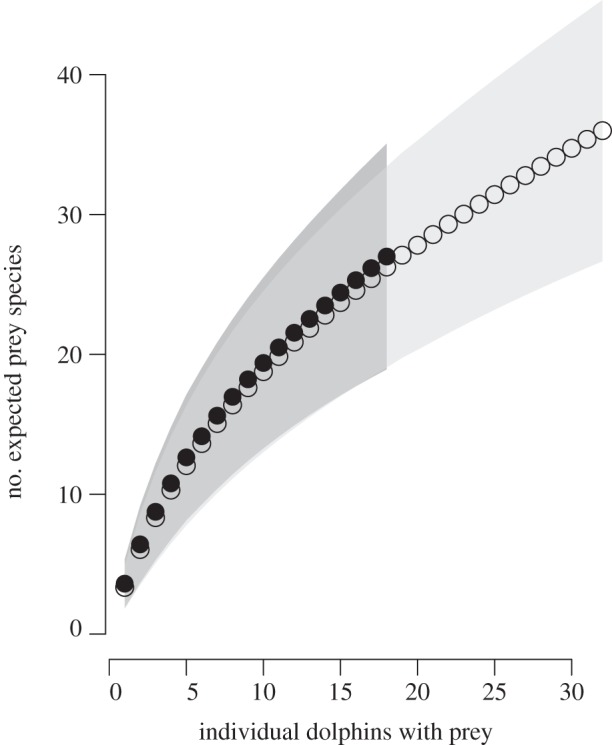
Mau Tau rarefaction curves and 95% CIs comparing prey diversity discovery between methods (SCA, open circles and light grey; DNA, closed circles and dark grey).

There were 12 MOTU taxonomic assignments not represented in SCA data, with seven novel to MOTU taxa, excluding possible overlaps in familial MOTU identifications (table 1). There were 21 SCA identifications not present in the MOTU taxonomic assignments, with 14 novel to SCA taxa, excluding possible overlaps in familial identifications (table 1). Only four and one of the novel prey taxa for the SCA and MOTU prey, respectively, were detected in more than one individual. Excepting the *Mugil gyrans/curema* MOTU, all species and genera identifications with an FOC > 10% from either dataset were represented in the other dataset at statistically similar FOC's (figure 1*a*) and proportional amounts (figure 1*b*). There was no difference in prey diversity accumulation between SCA and DNA methods, suggesting the differences in prey composition between datasets was partly due to sampling effort (figure 2). ANOSIM showed no difference between the two datasets for both prey presence/absence (Global *R* = −0.01; *p* = 0.54) and percent composition (Global *R* = −0.032; *p* = 0.73).

## 4. Discussion

Stomach contents analysis (SCA) is an important technique for top predator ecology. However, sampling dead animals opportunistically may result in biases caused by animal health and/or cause of death, producing unrepresentative but ecologically important structuring in the sample. Necropsies from many stranded cetaceans reveal evidence of poor health, interactions with fishing gear, or the cause of death is unknown (e.g. this study: see electronic supplementary material). In these cases, uncertainty of the representative nature of SCA remains.

This study quantitatively compared species-level diet information from free-ranging cetaceans with that inferred from stranded animals. Methodological constraints included the limited sample sizes and the potential incongruence between calculated diet metrics and consumed prey biomass. Given the agreement between back-calculated prey biomass and FOC in this population [1] and that quantitative prey signatures are detectable in controlled DNA-based diet studies [12,17], we think the latter constraint is addressed. Moreover, considering the different limitations and assumptions of each method and strong similarity between datasets, the simplest explanation of our results is that prey of stranded animals represents the forage of live animals with the corollary that, on average, the cause of death or stranding does not appear to significantly bias pre-stranding foraging in this population. This validation of SCA is important as management decisions and further work can be based on SCA results. Early SCA work on this population indicated that: (i) *Lagodon rhomboides*, a seagrass associated fish, was an important resource and (ii) the prevalence of soniferous fish in diets relative to their abundance may reflect a foraging strategy where dolphins listen for prey noise to seek prey, instead of actively using sonar (the ‘passive listening hypothesis’; [1]). These concepts have been important for current dolphin research [2,9] and are further supported by the results we present here.

Another striking aspect of the congruence between these datasets is the different time scales over which samples were collected. The temporal resolution of both methods is similar and probably represents the forage of individuals from up to ≈48 hours prior to death or sampling. The SCA data thus represent the average diet of stranded dolphins sampled sparsely over 22 years in all seasons, whereas the molecular data represent a one week snapshot of the living population's diet in two consecutive summers. The similarity of the two datasets, in combination with this population's restricted range and seagrass associations [1,2] suggests that the long-term average diet represents the summer diet of the population, which aids interpretation of drivers of habitat use and prey selection in relation to seasonal environmental variation.

This work has validated SCA as being representative of the diet of healthy free-ranging individuals for this inshore cetacean population. Populations that spend considerable time offshore may still suffer sampling bias owing to carcass availability and questions of the representative nature of these samples remain. However, these findings are likely to extend to other populations of *Tursiops truncatus* and other cetaceans with restricted inshore ranges and should aid interpretation of SCA results in these situations.

## References

[1] Barros NB Wells RS 1998 Prey and feeding patterns of resident bottlenose dolphins (*Tursiops truncatus*) in Sarasota Bay, Florida. J. Mammal. 79, 1045–1059 10.2307/1383114 (doi:10.2307/1383114)

[2] Berens McCabe EJ Gannon DP Barros NB Wells RS 2010 Prey selection by resident common bottlenose dolphins (*Tursiops truncatus*) in Sarasota Bay, Florida. Mar. Biol. 157, 931–942 10.1007/s00227-009-1371-2 (doi:10.1007/s00227-009-1371-2)

[3] Tollit DJ Pierce GJ Hobson KA Bowen WD Iverson SJ 2010 Diet. In Marine mammal ecology and conservation: a handbook of techniques (eds Boyd IL Bowen WD Iverson SJ), pp. 191–221 New York, NY: Oxford University Press

[4] Santos MB Clarke MR Pierce GJ 2001 Assessing the importance of cephalopods in the diets of marine mammals and other top predators: problems and solutions. Fish. Res. 52, 121–139 10.1016/S0165-7836(01)00236-3 (doi:10.1016/S0165-7836(01)00236-3)

[5] Barros NB Clarke MR 2002 Diet. In Encyclopedia of marine mammals (eds Perrin WF Wursig B Thewissen JGM), pp. 323–327 San Diego, CA: Academic Press

[6] Dunshea G 2009 DNA-based diet analysis for any predator. PLoS ONE 4, e5252 10.1371/journal.pone.0005252 (doi:10.1371/journal.pone.0005252) 19390570 PMC2668750

[7] Jarman SN Deagle BE Gales NJ 2004 Group-specific polymerase chain reaction for DNA-based analysis of species diversity and identity in dietary samples. Mol. Ecol. 13, 1313–1322 10.1111/j.1365-294X.2004.02109.x (doi:10.1111/j.1365-294X.2004.02109.x) 15078466

[8] Symondson WOC 2002 Molecular identification of prey in predator diets. Mol. Ecol. 11, 627–641 10.1046/j.1365-294X.2002.01471.x (doi:10.1046/j.1365-294X.2002.01471.x) 11972753

[9] Gannon DP Barros NB Nowacek DP Read AJ Waples DM Wells RS 2005 Prey detection by bottlenose dolphins, *Tursiops truncatus*: an experimental test of the passive listening hypothesis. Anim. Behav. 69, 709–720 10.1016/j.anbehav.2004.06.020 (doi:10.1016/j.anbehav.2004.06.020)

[10] Wells RS 2004 Bottlenose dolphins as marine ecosystem sentinels: developing a health monitoring system. EcoHealth 1, 246–254 10.1007/s10393-004-0094-6 (doi:10.1007/s10393-004-0094-6)

[11] Munch K Boomsma W Huelsenbeck JP Willerslev E Nielsen R 2008 Statistical assignment of DNA sequences using Bayesian phylogenetics. Syst. Biol. 57, 750–757 10.1080/10635150802422316 (doi:10.1080/10635150802422316) 18853361

[12] Dunshea G 2012 Ecological diagnostics for marine mammals: appraisal of molecular-based methods for dietary and age estimation. PhD thesis, University of Tasmania, Australia

[13] Wright BE 2010 Use of chi-square tests to analyze scat-derived diet composition data. Mar. Mammal Sci. 26, 395–401 10.1111/j.1748-7692.2009.00308.x (doi:10.1111/j.1748-7692.2009.00308.x)

[14] Colwell RK 2005 EstimateS: statistical estimation of species richness and shared species from samples, version 7.5. See http://purl.oclc.org/estimates

[15] Clark KR Gorley RN 2006 PRIMER v6: User Manual/Tutorial. PRIMER-E, Plymouth. See http://www.primer-e.com/

[16] Dunshea G Barros NB Wells RS Gales NJ Hindell MA Jarman SN 2008 Pseudogenes and DNA based diet analyses; a cautionary tale from a relatively well sampled predator/prey system. Bull. Entomol. Res. 98, 239–248 10.1017/S0007485308005993 (doi:10.1017/S0007485308005993) 18439341

[17] Bowles E Schulte PM Tollit DJ Deagle BE Trites AW 2011 Proportion of prey consumed can be determined from faecal DNA using real-time PCR. Mol. Ecol. Resour. 11, 530–540 10.1111/j.1755-0998.2010.02974.x (doi:10.1111/j.1755-0998.2010.02974.x) 21481211

